# Increased Risk Proneness or Social Withdrawal? The Effects of Shortened Life Expectancy on the Expression of Rescue Behavior in Workers of the ant *Formica cinerea* (Hymenoptera: Formicidae)

**DOI:** 10.1007/s10905-017-9647-8

**Published:** 2017-11-04

**Authors:** Krzysztof Miler, Beata Symonowicz, Ewa J. Godzińska

**Affiliations:** 1 Institute of Environmental Sciences, Jagiellonian University, Gronostajowa St. 7, 30-387 Kraków, Poland; 2 Laboratory of Ethology, Department of Neurophysiology, Nencki Institute of Experimental Biology of the Polish Academy of Sciences, Pasteur St. 3, 02-093 Warszawa, Poland

**Keywords:** Life expectancy, rescue behavior, prosocial behavior, carbon dioxide poisoning, antlion, *Formica cinerea*

## Abstract

In social insects behavioral consequences of shortened life expectancy include, among others, increased risk proneness and social withdrawal. We investigated the impact of experimental shortening of life expectancy of foragers of the ant *Formica cinerea* achieved by their exposure to carbon dioxide on the expression of rescue behavior, risky pro-social behavior, tested by means of two bioassays during which a single worker (rescuer) was confronted with a nestmate (victim) attacked by a predator (antlion larva capture bioassay) or immobilized by an artificial snare (entrapment bioassay). Efficacy of carbon dioxide poisoning in shortening life expectancy was confirmed by the analysis of ant mortality. Rescue behavior observed during behavioral tests involved digging around the victim, transport of the sand covering the victim, pulling the limbs/antennae/mandibles of the victim, direct attack on the antlion (in antlion larva capture tests), and snare biting (in entrapment tests). The rate of occurrence of rescue behavior was lower in ants with shortened life expectancy, but that effect was significant only in the case of the entrapment bioassay. Similarly, only in the case of the entrapment bioassay ants with shortened life expectancy displayed rescue behavior after a longer latency and devoted less time to that behavior than ants from the control groups. Our results demonstrated that in ant workers shortened life expectancy may lead to reduced propensity for rescue behavior, most probably as an element of the social withdrawal syndrome that had already been described in several studies on behavior of moribund ants and honeybees.

## Introduction

Risk proneness and individual life expectancy of social insect workers are strongly connected. Such workers as a rule engage in intranidal tasks at the start of their adult life and then switch to more risky extranidal activities as they age (Wilson 1971; Hölldobler and Wilson 1990, 2009). Delayed performance of risky tasks enhances mean worker longevity, which is beneficial for the colony (Jeanne 1986; O’Donnell and Jeanne 1995; Woyciechowski and Kozłowski 1998; Tofilski 2002, 2009). Many studies provided evidence that participation in risky tasks involved in foraging or colony defense is indeed delayed until late in worker life (for examples in honeybees, ants, and wasps see Winston and Katz 1981; Schmid-Hempel and Schmid-Hempel 1984; O’Donnell and Jeanne 1992). Other studies demonstrated that experimental shortening of worker life expectancy accelerates the transition from intranidal tasks to foraging in ants (Moroń et al. 2008) and honeybees (Woyciechowski and Moroń 2009). Such acceleration of worker behavioral development was observed irrespectively of experimental treatment applied to reduce worker life expectancy: poisoning with carbon dioxide, physical harm, or parasitic infection (Moroń et al. 2008; Woyciechowski and Moroń 2009). Ants with life expectancy shortened either by poisoning with carbon dioxide, or by removal of propodeal spines, were also more prone to engage in foraging under more risky conditions (Moroń et al. 2012).

Decreased life expectancy impacts not only risk proneness, but also other aspects of worker behavior. In particular, injured and/or infected ants were often observed to reduce contacts with nestmate workers and/or brood by avoidance of interactions, loss of attraction to social cues and spatial separation (staying away from brood chambers or even leaving the nest altogether) (Hölldobler and Wilson 1990; Ugelvig and Cremer 2007; Aubert and Richard 2008; Heinze and Walter 2010; Bos et al. 2011; Diez et al. 2015; Leclerc and Detrain 2017). Similar reduction of caregiving behavior and/or altruistic self-removal of health-compromised workers was also documented in the honeybees (Wang and Moeller 1970; Shimanuki et al. 1994; Kralj and Fuchs 2006; Rueppell et al. 2010). In natural conditions shortened life expectancy often occurs due to transmittable sickness. Therefore, it was argued that social withdrawal of moribund individuals may benefit the colony by limiting disease transmission (Evans 1982; Heinze and Walter 2010; Rueppell et al. 2010; Bos et al. 2011). However, it should also be noted that infected ant workers frequently remain not attacked and even not avoided by their nestmates who respond to them by hygienic behavior (Ugelvig and Cremer 2007; Aubert and Richard 2008; Heinze and Walter 2010; Konrad et al. 2012). Indeed, an opposite phenomenon – increased propensity for social interactions – is sometimes observed (de Souza et al. 2008; Hamilton et al. 2011). Increased rate of contacts with infected individuals is beneficial both for the infected individuals who survive longer in groups than in isolation (Houghes et al. 2002; Walker and Houghes 2009; Heinze and Walter 2010) and for their nestmates who increase their resistance against the infection thanks to activation of the immune system (Ugelvig and Cremer 2007; Aubert and Richard 2008; Konrad et al. 2012). Clearly, the prevention of disease spread in insect societies may involve many tactics, some of them seemingly opposite.

Ant rescue behavior provides an excellent opportunity for studying the relationship between life expectancy and both risk proneness and social withdrawal. Rescue behavior belongs to the more general category of pro-social behavior, i.e. behavior which provides benefits for another individual(s) (Vasconcelos et al. 2012). Rescue behavior is defined as a social interaction during which one individual – the victim – is endangered (in risk of severe physical harm), and another individual – the rescuer – places itself at risk of endangerment by engaging in rescue attempt. The behavior of the rescuer must also be generally suited to the circumstances, and not inherently rewarding or beneficial to the rescuer (Hollis and Nowbahari 2012). Behavior patterns involved in ant rescue behavior were investigated in several experimental studies by means of two bioassays: the antlion larva capture bioassay (Czechowski et al. 2002; Taylor et al. 2013; Miler 2016; Miler et al. 2017) and the entrapment bioassay (Nowbahari et al. 2009, 2012, 2016; Hollis and Nowbahari 2013; Duhoo et al. 2017; Miler and Kuszewska 2017; Miler et al. 2017). Antlion larvae display quite sophisticated hunting techniques: they construct pitfall traps to catch insect prey, especially ants (Hollis et al. 2015; Hollis 2017). Such a pitfall trap acts as the main element of experimental setting in the antlion larva capture bioassay. Although the antlion larva capture bioassay is more closely related to environmental conditions encountered by some ants, the entrapment bioassay is used more frequently, possibly because it is more easily standardized. Recently, rescue behavior was also observed in termite-hunting ants *Megaponera analis* which reduce raid mortality by helping nestmates injured during fights with termite soldiers (Frank et al. 2017).

The aim of the present study was to assess the impact of shortened life expectancy on the expression of rescue behavior in ant workers. On the basis of the results of earlier research that demonstrated a close association between life expectancy and risk proneness, we predicted that individuals with shortened life expectancy should be more prone to engage in rescue behavior than ants with longer life expectancy, as such soon-to-die individuals are, anyway, of low value for their colony, serving as the so-called “disposable caste” (Porter and Jorgensen 1981): if they perish while engaging in a risky task, they can be sacrificed at a relatively low cost. However, as rescue behavior involves a social contact between the victim and the rescuer, it is also possible that such a social interaction will be avoided by individuals with shortened life expectancy as a part of behavioral syndrome of social withdrawal. To differentiate between these alternatives, we investigated the impact of higher versus lower life expectancy on the occurrence and characteristic traits of rescue behavior of foragers of the ant species *Formica cinerea*. We used carbon dioxide poisoning as means of shortening the life expectancy of workers, a method already used in earlier studies on the effects of shortened life expectancy on ant behavior (Moroń et al. 2008, 2012; Heinze and Walter 2010), including a study investigating the effects of shortened life expectancy of the victims on behavior of potential rescuers in workers of *F. cinerea* tested by means of antlion larva capture bioassays (Miler 2016).

## Materials and Methods

About 6000 active *F. cinerea* foragers from three different colonies (about 2000 workers from each colony) were hand-picked in the field near Klucze (close to Błędowska Desert in southern Poland) along with about 300 co-occurring antlion larvae (*Myrmeleon bore*). In the laboratory, both ants and antlions were allowed to acclimatize to laboratory conditions for 48 h (constant ambient temperature of 24 °C, 40–60% RH and 12:12 L:D cycle). Antlions were kept separately in plastic cups (7 cm in diameter, 15 cm high) half filled with dry sand. They were not fed during the short period between their capture and the start of behavioral tests (2 days). Ants from different colonies were kept in separate open plastic boxes (25 × 17 × 10 cm) with walls coated with Fluon ® (PTFE) (oobleck, Warsaw, Poland), a substance providing silky smooth surface and, hence, commonly used in myrmecological research to prevent the ants from escaping from artificial nests. Ants were provided with water and sucrose solution *ad libitum*. After acclimatization, each colony fragment was split into halves (a control half and an experimental one) which were then kept in separate open plastic boxes (25 × 17 × 10 cm) with fluon-covered walls. Both experimental and control boxes with ants were then put in impermeable plastic bags. Bags containing control boxes were sealed immediately afterwards, whereas bags containing experimental boxes were first inflated with ~100% carbon dioxide for about 5 min, and only then sealed. All plastic bags were removed after 1.5 h. Immediately after this treatment, 50 ants were taken from the control half and the experimental half of each colony and put in separate open plastic boxes (25 × 17 × 10 cm) with fluon-covered walls. These ants were provided with water and saturated sucrose solution *ad libitum*, and their mortality was checked daily until all were dead, starting on the next day after CO_2_ treatment. Each dead ant was given a score corresponding to the day of its death, the survivorship score (e.g. an ant that died on the day 5 was given a score of 5). Thus, groups of ants which experienced accelerated death would obtain lower survivorship scores than control groups.

Behavioral tests started on the next day after CO_2_ treatment. Two types of bioassays were performed: antlion larva capture and entrapment. For antlion larva capture tests, we used the cups in which the antlions were kept, using only antlions with fully functional pitfall traps, which were ready to capture prey. In each test, a randomly chosen forager, taken from the control group by means of forceps, was dropped into the antlion pit. Immediately after the ant was captured by the antlion, a potential rescuer (from either the control group or the experimental one) was introduced into the cup (but not into the pit).

For entrapment tests, we used cups of the same size as in the case of tests with antlions, also half filled with dry sand. A randomly chosen forager taken from the control group was tied by means of nylon thread (0.22 mm in diameter) passing over the petiole to a small, 1.5 cm in diameter piece of filter paper, and placed inside the cup in such a way that its body was visible, but the filter paper was covered with a thin layer of sand (sprinkled from above). Immediately after, a potential rescuer (from either control or experimental group) was introduced into the cup (but not in direct proximity to the victim, at a distance of at least 1.5 cm from the entrapped ant). In both bioassays, the tests started immediately after the potential rescuer was introduced, and lasted for 3 min. Rare cases of tests which lasted shorter than 3 min due to antlion behavior, i.e. victim burial under the sand surface or release of the victim, were discarded. We noted whether rescue behavior occurred, and if so, we noted the latency to the first episode of rescue behavior and the total duration of rescue. Similarly as in the previous experiments (Miler 2016; Miler and Kuszewska 2017; Miler et al. 2017), digging around the victim, pulling at its limbs/antennae/mandibles, transport of the sand covering the victim, snare biting in entrapment tests, and direct attack on the antlion in antlion larva capture tests were considered by us as the main subcategories of rescue behavior. Our final dataset included the results of 30 tests for each group (control and experimental) in each bioassay in each of three colonies (in total, 360 tests). Each ant was used only once. Antlions which were ready to capture prey were also used only once. All tests with the ants taken from the same colony fragments (in total 120 tests) were performed on the single day, the day after the experimental treatment, in a counterbalanced manner (one control test in the entrapment bioassay, one experimental test in the entrapment bioassay, one control test in the antlion larva capture bioassay, one experimental test in the antlion larva capture bioassay, and so on). Three colony fragments acting as a source of workers for the experiment were used one after another within a single week. Each colony fragment was collected in the field, acclimatized, treated and tested, and then the next one underwent the same procedure. Data obtained for two bioassays were analyzed separately. We used the two-tailed Fisher’s Exact Test (FET) for detecting inter-group differences in the rate of occurrence of rescue behavior. Data on mortality were analyzed by means of Generalized Linear Mixed Model (GLMM), with group as a fixed factor and colony as a random factor, using a loglink function and Poisson error distribution (dependent variable: the survivorship score). Data on the latency to rescue behavior and the duration of rescue behavior were first log-transformed and then analyzed by means of GLMM with group as a fixed factor and colony as a random factor, using an identity function and normal error distribution (dependent variables: the latency to the first episode of rescue behavior and the total duration of rescue behavior). Only the tests in which rescue behavior occurred were taken into account in analyses of the latency to rescue behavior and the total duration of rescue behavior. Statistical analyses were performed in SPSS Statistics 21 (IBM, Warsaw, Poland). Latency to rescue and total duration of rescue are linked in our experimental design, i.e. duration data are biased because total duration depends to some extent on latency due to fixed test time. Thus, we adjusted the values of total duration of rescue behavior by dividing the total duration of rescue by the total duration of the test (180 s) reduced by the latency to the first episode of rescue. To avoid analyzing fraction values, results of these calculations were in each case multiplied by a hundred and rounded to obtain whole numbers. Identical adjustment of total duration was performed previously in the analysis of the results of a similar experiment (Miler 2016).

## Results

**Fig. 1:**
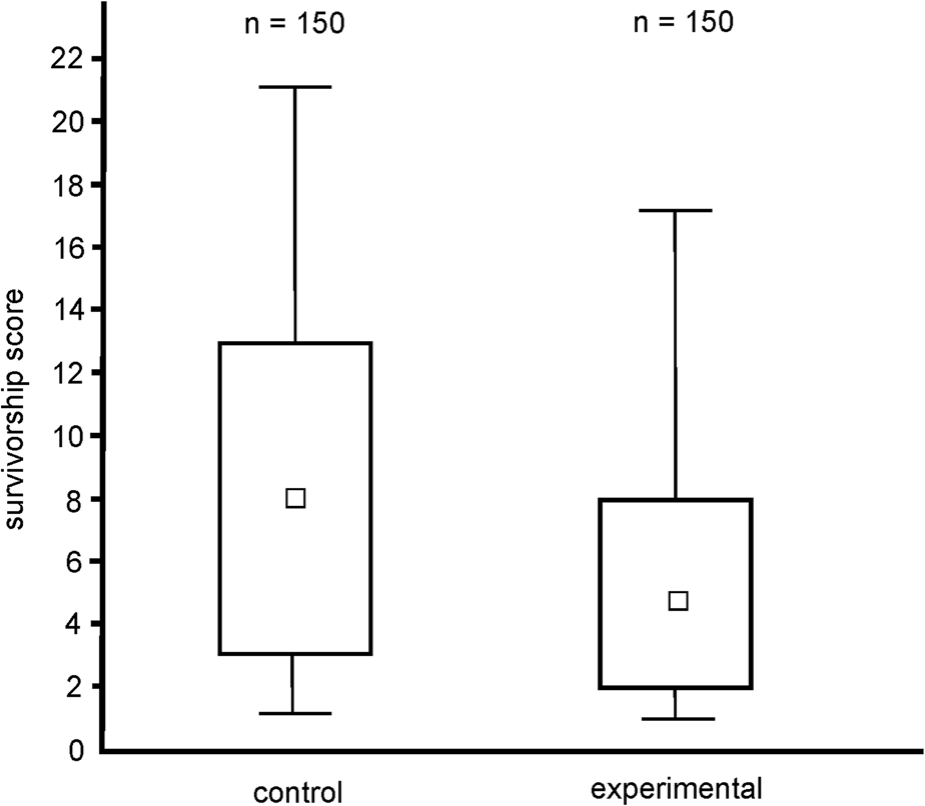
Comparison of survivorship of workers of the ant *Formica cinerea* in control (untreated) and experimental (subjected to CO_2_ poisoning) group (*n* = 150 in each group). Survivorship score of each worker denotes the number of days from the start of the experiment until its death. Squares, boxes and whiskers: medians, quartiles and range, respectively. Statistics: GLMM; F_1,298_ = 88.210, *p* < 0.0001

Mortality analysis confirmed that our experimental treatment (CO_2_ poisoning) was effective in shortening life expectancy of captive foragers of *F. cinerea* (Fig. 1).

**Fig. 2:**
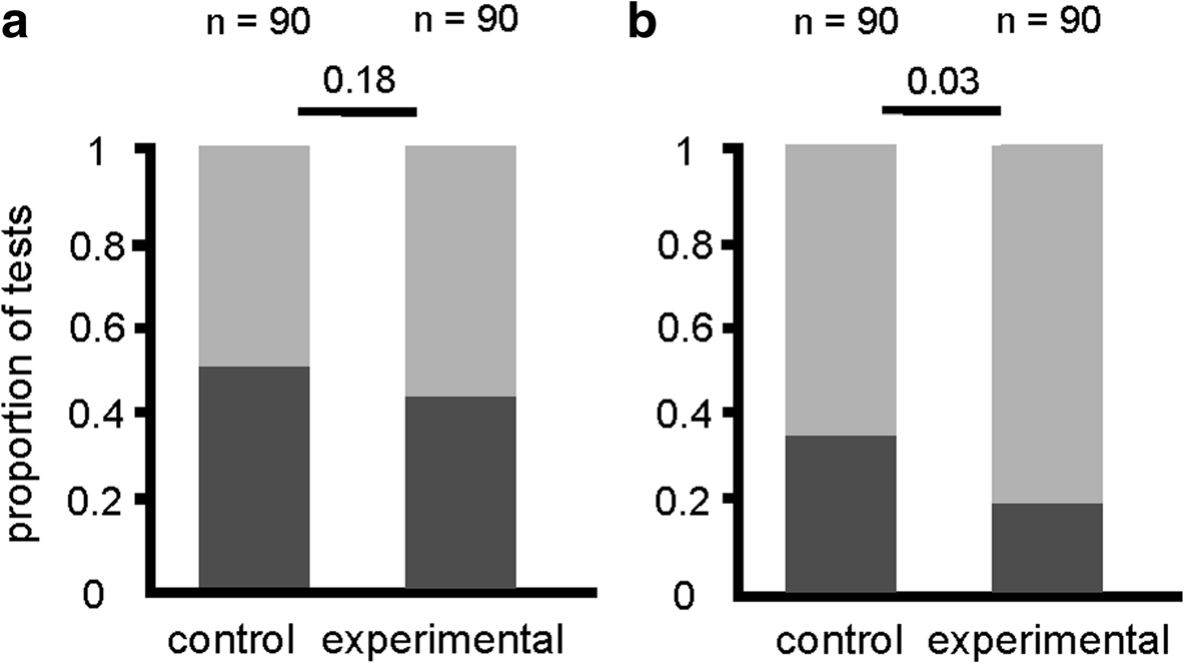
The proportion of tests during which rescue behavior was observed (dark grey bars) and not observed (light grey bars) in the control (untreated) and the experimental (subjected to CO_2_ poisoning) group of workers of the ant *Formica cinerea* (*n* = 90 in each group)*.* **a** Antlion larva capture bioassay; **b** Entrapment bioassay. Statistics: two-tailed Fisher’s Exact Test; *p* values for differences between groups are indicated above the bars

**Fig. 3:**
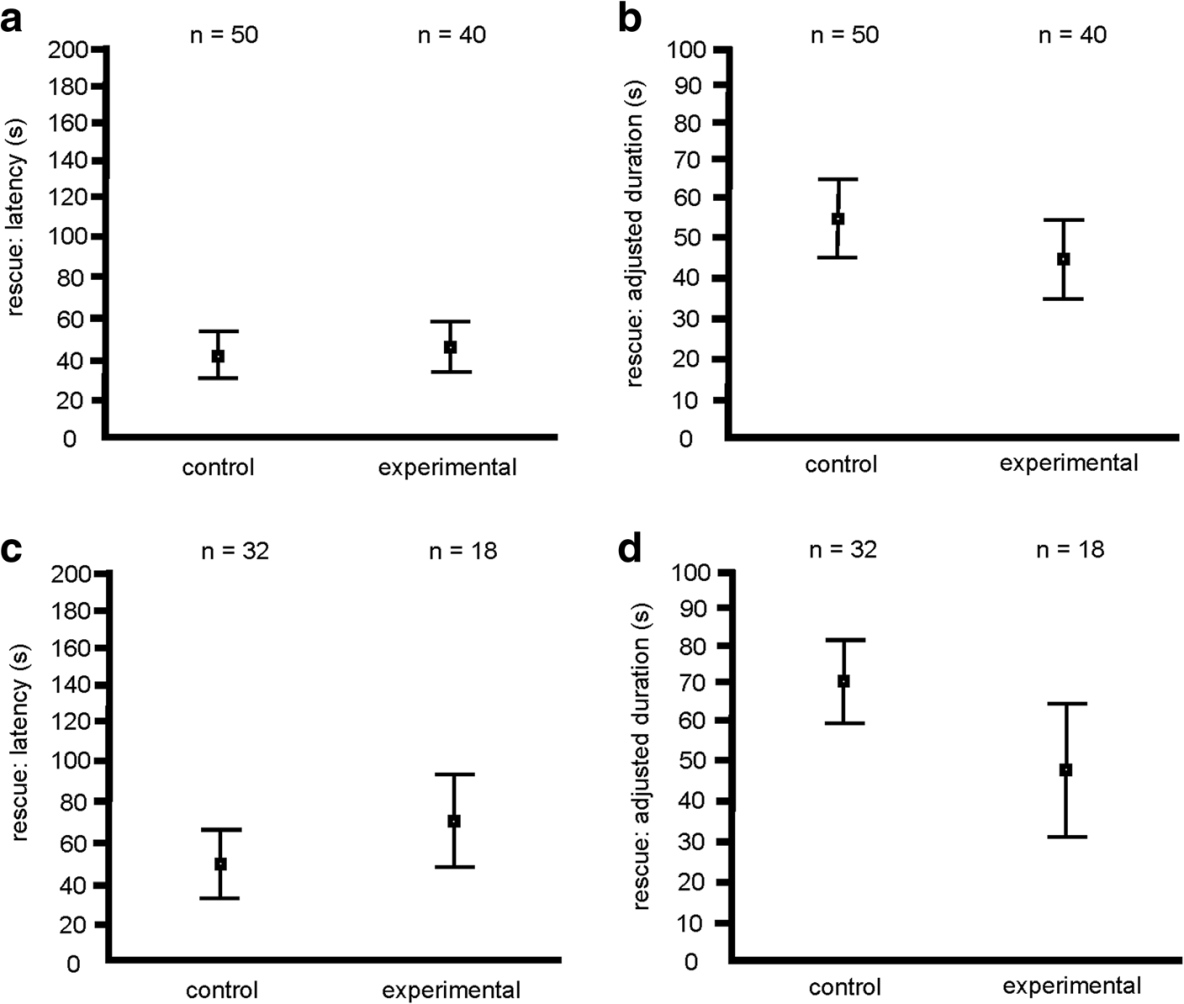
Latency to the first episode of rescue and the adjusted total duration of rescue behavior in the control (untreated) and the experimental (subjected to CO_2_ poisoning) groups of workers of the ant *Formica cinerea*. Antlion larva capture bioassay: **a** latency to the first episode of rescue behavior (GLMM: F_1,88_ = 0.531, *p* = 0.468); **b** adjusted total duration of rescue behavior (GLMM: F_1,88_ = 2.306, *p* = 0.132). Entrapment bioassay: **c** latency to the first episode of rescue behavior (GLMM: F_1,48_ = 6.199, *p* = 0.016); **d** adjusted total duration of rescue behavior (GLMM: F_1,48_ = 4.295, *p* = 0.044). Squares and whiskers: mean and CI, respectively

In the case of antlion larva capture bioassay, the rate of occurrence of rescue behavior did not differ significantly between the ants from the control group and the experimental group (Fig. 2a). The latency to the first episode of rescue behavior and the total adjusted duration of rescue did not differ between the control group and the experimental groups (Fig. 3a, b). In the case of entrapment bioassay, however, the rate of occurrence of rescue behavior was significantly lower in the ants with shortened life expectancy than in the ants from the control group (Fig. 2b). Also, ants with shortened life expectancy were found to start to rescue the victim after a significantly longer latency (Fig. 3c) and to devote significantly less time to rescue behavior (Fig. 3d) than ants from the control group.

## Discussion

The comparison of mortality of workers subjected or not subjected to CO_2_ poisoning revealed that this treatment did indeed shorten life expectancy of workers of *F. cinerea*. This result is in concordance with the results of other studies on the effects of exposure to carbon dioxide on longevity of ant workers (Moroń et al. 2008, 2012; Miler 2016) and other insects (Nicolas and Sillans 1989; Gannon et al. 2001). However, the exact physiological and behavioral effects induced by CO_2_ poisoning are not known. We cannot exclude that such treatment may also provoke effects other than accelerated ageing, such as, for instance, modifications of the functioning of the nervous system related to irreversible damage exerted on nerve membranes (Gannon et al. 2001; Moroń et al. 2008). The effect of CO_2_ poisoning in the form of e.g. withdrawal from some forms of non-social behavior would be especially important in the context of the present study and is worth further study. Indeed, *Drosophila* flies were recently demonstrated to experience motor impairment following CO_2_ poisoning (Bartholomew et al. 2015). In ants, however, no long-term motor disturbances following CO_2_ poisoning were so far reported, and in our present study none were obvious 24 h post-treatment. In future studies investigating the effects of shortened life expectancy on the expression of ant behavior it could be advantageous to apply not only CO_2_ poisoning, but also other treatments used to shorten life expectancy in ants, such as physical injury (Moroń et al. 2008, 2012) and/or infection (Leclerc and Detrain 2017). This will allow us to estimate whether and to what degree behavioral modifications observed in individuals that had been subjected to CO_2_ poisoning are related solely to that specific treatment, and which of these modifications can be observed also in individuals that had been subjected to different treatments used to shorten worker life expectancy.

Contrary to our predictions, shortening of life expectancy was not accompanied by increased propensity for risky altruism in workers of the ant species *F. cinerea.* During entrapment bioassays workers with shortened life expectancy engaged in rescue behavior less frequently than ants from the control group (Fig. 2b), started to display that behavior after a longer delay from the start of the test (Fig. 3c), and devoted less time to their attempts to free the victim (Fig. 3d).

Decreased propensity for rescue behavior observed in ants with shortened life expectancy may represent an element of the so-called social withdrawal syndrome, i.e. self-isolation of moribund individuals, which is well documented in ants (Hölldobler and Wilson 1990; Ugelvig and Cremer 2007; Aubert and Richard 2008; Heinze and Walter 2010; Bos et al. 2011; Diez et al. 2015; Leclerc and Detrain 2017) and in honeybees (Wang and Moeller 1970; Shimanuki et al. 1994; Kralj and Fuchs 2006; Rueppell et al. 2010), and is usually considered to act as one of the tactics of defense against disease spread. Other tactics employed by social insects in the prevention of infectious diseases are not based on physical separation of sick nestmates from healthy ones, but, on the contrary, involve intense social contacts between sick and healthy individuals (see e.g. Aubert and Richard 2008; Konrad et al. 2012). However, although infected individuals may participate in intense social contacts, they usually behave passively, accepting contact carried out by their nestmates, but avoiding to initiate social interactions (Ugelvig and Cremer 2007; Aubert and Richard 2008; Bos et al. 2011; Konrad et al. 2012). In other words, passive acceptance of social contact and withdrawal from more active forms of interactions are not mutually exclusive and may co-occur. As ant rescue behavior requires very active participation in a specific social interaction, decreased propensity for that behavior shown by workers with shortened life expectancy may well represent an element of the social withdrawal syndrome.

According to the working hypothesis commonly held in the research on ant rescue behavior, an endangered individual is sending some kind of a call for help that elicits rescue behavior in its nestmates and sometimes also non-nestmates. Lower propensity for engaging in rescue behavior observed in moribund individuals may result from their lower sensitivity to such calls for help, i.e. some pheromone(s) and/or vibroacoustic signals emitted by the victim (Jackson and Morgan 1993; Hunt and Richard 2013; Frank et al. 2017), which are much more likely to play the role of calls for help than, for instance, cuticular hydrocarbons, usually indicated as major factors involved in the mediation of interactions between nestmates in social insect colonies (Richard and Hunt 2013). Emission of vibratory signals by restrained ants has been documented in many studies (Markl 1965; Masters 1979; Stuart and Bell 1980; Rauth and Vinson 2006), and it has even been hypothesized that stridulation first evolved among the ants to alert nestmates of burial and need for rescue (a hypothesis now rejected; Golden and Hill 2016). Frank et al. (2017) also demonstrated that artificially injured workers of *Megaponera analis* made unable to stridulate were still rescued by their nestmates. Moreover, stridulatory organs are absent in the ants from the subfamily Formicinae (Czechowski et al. 2002; Golden and Hill 2016), so calls for help emitted by the tested workers of *F. cinerea* could not have involved vibroacoustic signals.

Identification of the exact nature of ant calls for help is an interesting issue which remains very poorly studied (Frank et al. 2017; Miler and Kuszewska 2017). Moreover, the results of the experiments devoted to that question suggest strongly that the exact nature of chemical signals acting as calls for help may differ between various ant species and/or various contexts in which ants engage in rescue behavior. Thus, Frank et al. (2017) found out that in workers of termite-hunting ant *Megaponera analis* rescue behavior consisting of carrying the nestmate back to the nest may be triggered by two compounds present in the mandibular gland reservoirs. However, Miler and Kuszewska (2017) demonstrated that secretions of mandibular glands are not involved in the elicitation of rescue behavior in workers of *Formica cinerea* during entrapment bioassays. The question of possible modifications of sensitivity to the calls for help as a function of individual life expectancy of the potential rescuers was so far never investigated.

In this study behavioral effects of shortened life expectancy were significant only in the case of entrapment bioassay. This context-dependence of behavioral effects of experimental treatment might have been related to differences in risk level faced by the potential rescuers in two bioassays used in this study. It seems probable that calls for help emitted by imperiled individuals to elicit rescue behavior in their nestmates (Frank et al. 2017; Miler and Kuszewska 2017) may differ depending on the context in which they are emitted, and, in particular, may show risk level related differences. It is highly likely that the participation in the antlion larva capture bioassay may be more alarming for the victim than the participation in the entrapment bioassay due to more immediate threat related to the presence of the predator. Note that in the antlion larva capture bioassays the latency to the first episode of rescue behavior was generally low in both groups (Fig. 3a) and that rescue behavior was observed in a larger number of tests than in the case of entrapment bioassay (Figs. 2 and 3). This may indicate that during antlion larva capture bioassays the call for help signals were stronger and/or released by the imperiled captured individuals after a shorter delay than during entrapment bioassays. As a consequence, more efficient triggering of rescue behavior partly masked the differences between the control and the experimental group related to different life expectancy. Differences between rescue behavior observed in different bioassays are documented also by other studies. Indeed, in an earlier study testing the performance of normal *F. cinerea* foragers in the antlion larva capture bioassay (Miler 2016) the mean latency from the start of the test to the onset of rescue behavior was similarly low as in the present study (42 s, as compared to 44 s in the present study). In another study (Miler and Kuszewska 2017), normal *F. cinerea* foragers tested by means of the entrapment bioassay started to engage in rescue behavior after a longer delay, with mean latency value of 97 s (in the present study this value reached 54 s). In conclusion, it seems plausible to suppose that absence of life expectancy effects in the antlion larva capture bioassay may result from the fact that this context is more alarming for both the victims and the potential rescuers.

The observed differences between the behavior of workers of *F. cinerea* during two types of bioassays may also be related to the fact that the behavior of antlion larvae induced additional variance in performance of potential rescuers that could mask the effects of life expectancy. A similar explanation was offered recently in a different study on rescue behavior of *Iridomyrmex anceps* ants, which showed rescue behavior in the context of entrapment bioassays but not in the context of antlion larva capture bioassays, possibly due to antlion behavior (Miler et al. 2017). Antlions inject paralytics into their prey (Matsuda et al. 1995; Yoshida et al. 1999), and there is the possibility that some ants are paralyzed faster than others, thus adding variance to the behavior of potential rescuers. We also did not investigate the possible effects of the instar stage of our antlion larvae, and instar stage, at least in some antlion species, is known to affect pitfall trap characteristics (Griffiths 1986), feeding behavior (Alcalay et al. 2014), and plausibly also paralytic capacity.

In social Hymenoptera worker behavioral specialization in intranidal versus extranidal tasks depends not only on worker age, with young workers participating in intranidal tasks and switching to extranidal activities as they age (Wilson 1971; Hölldobler and Wilson 1990, 2009), but also on worker physiological maturation (McDonald and Topoff 1988; Fénéron et al. 1996), and on worker life expectancy which is not strictly correlated with worker age (Jeanne 1986; O’Donnell and Jeanne 1995; Woyciechowski and Kozłowski 1998; Tofilski 2002, 2009). The effect of behavioral specialization of ant workers in intranidal vs extranidal tasks on their ability to receive and give rescue behavior was investigated in ants from the species *Cataglyphis cursor* in two contexts: rescue of adult workers (Nowbahari et al. 2012) and rescue of very young, newly eclosed ants, the so called callows (Nowbahari et al. 2016). As demonstrated by these studies, callows of *C. cursor* were rescued in an equally efficient way by nurses and by foragers (Nowbahari et al. 2016). However, in the experiments with older individuals of that species foragers proved to be the most efficient at both administering help to the victims and obtaining it from the rescuers, probably due to physiological tuning resulting from ecological relevance of rescue behavior for specialization in extranidal tasks (Nowbahari et al. 2012).

In this study, we used as subjects only active foragers collected in the field late during season, which implies that life expectancy was relatively low in the case of all ants used as subjects. In these relatively old foragers of *F. cinerea* exposure to CO_2_ for 1.5 h led to alterations of rescue behavior suggesting the induction of social withdrawal. Relatively old foragers of that species subjected to identical CO_2_ treatment were also reported to show reduced ability to induce rescue behavior in potential rescuers, which may also be interpreted in terms of social withdrawal (Miler 2016). However, the same treatment (exposure to CO_2_ for 1.5 h) applied to young workers of another ant species, *Myrmica scabrinodis,* was followed by their accelerated transition from less risky intranidal activities to more risky extranidal tasks (Moroń et al. 2008). The same treatment applied to relatively young foragers of *M. scabrinodis* was also followed by higher risk proneness of the treated workers during foraging (Moroń et al. 2012). Lastly, prolonged CO_2_ poisoning (80 h) applied to young workers of yet another ant species, *Temnothorax unifasciatus*, was followed by a dramatic form of social withdrawal (Heinze and Walter 2010). All these results taken together suggest that while shortened life expectancy results in increased risk proneness, further drop in life expectancy may lead to social withdrawal. As pointed out by Moroń et al. (2012), similar results were obtained also in the experiments with honeybees: short (0.33 h) exposure of young honeybee workers to carbon dioxide was followed by accelerated transition to foraging (Woyciechowski and Moroń 2009), but longer CO_2_ poisoning (1.75 h) was followed by social withdrawal of the treated bees (Rueppell et al. 2010).

To conclude, our study revealed that workers of *F. cinerea* with life expectancy shortened by CO_2_ poisoning are less prone to engage in rescue behavior. This behavioral modification might constitute an element of behavioral syndrome of social withdrawal, reduction of social contacts acting as one of the tactics of prevention of disease spread. Importantly, this effect was significant only in the case of one of two bioassays used in this study, the entrapment bioassay, plausibly reflecting high context-dependency of the propensity for rescue behavior in workers of *F. cinerea*.
